# Optogenetic dissection of amygdala functioning

**DOI:** 10.3389/fnbeh.2014.00107

**Published:** 2014-03-26

**Authors:** Ryan T. LaLumiere

**Affiliations:** Department of Psychology, University of IowaIowa City, IA, USA

**Keywords:** basolateral amygdala, central amygdala, memory, consolidation, fear, anxiety, channelrhodopsin

## Abstract

Studies of amygdala functioning have occupied a significant place in the history of understanding how the brain controls behavior and cognition. Early work on the amygdala placed this small structure as a key component in the regulation of emotion and affective behavior. Over time, our understanding of its role in brain processes has expanded, as we have uncovered amygdala influences on memory, reward behavior, and overall functioning in many other brain regions. Studies have indicated that the amygdala has widespread connections with a variety of brain structures, from the prefrontal cortex to regions of the brainstem, that explain its powerful influence on other parts of the brain and behaviors mediated by those regions. Thus, many optogenetic studies have focused on harnessing the powers of this technique to elucidate the functioning of the amygdala in relation to motivation, fear, and memory as well as to determine how the amygdala regulates activity in other structures. For example, studies using optogenetics have examined how specific circuits within amygdala nuclei regulate anxiety. Other work has provided insight into how the basolateral and central amygdala nuclei regulate memory processing underlying aversive learning. Many experiments have taken advantage of optogenetics’ ability to target either genetically distinct subpopulations of neurons or the specific projections from the amygdala to other brain regions. Findings from such studies have provided evidence that particular patterns of activity in basolateral amygdala (BLA) glutamatergic neurons are related to memory consolidation processes, while other work has indicated the critical nature of amygdala inputs to the prefrontal cortex and nucleus accumbens (NA) in regulating behavior dependent on those downstream structures. This review will examine the recent discoveries on amygdala functioning made through experiments using optogenetics, placing these findings in the context of the major questions in the field.

Despite its relatively small size in the human brain, the amygdala influences a wide variety of neural functions, especially those related to emotion and memory. As a result, considerable research has targeted this structure and investigated how it regulates different processes and interacts with other brain regions to do so. The development of optogenetics over the past decade has opened new doors for investigations into neural circuits, both on a systems-circuitry level and on a microcircuitry level, and into how those circuits govern behavior. The amygdala, with its rich history of research in neuroscience, has been a ripe target for optogenetic investigations and, thus, many studies have applied this technique to address deeper issues regarding amygdala functioning, providing new avenues of research into “old” questions about the amygdala. Because optogenetics permits stimulation and inhibition of specific projection pathways through illumination of opsin-expressing axons and axon terminals (LaLumiere, 2011; Yizhar et al., 2011), many studies have used this approach to explore specific circuits. Moreover, because opsin expression can be genetically targeted to distinct cell types, many studies have also taken advantage of this capability to control activity in specific classes of neurons to determine their functional roles. This review will explore the recent studies that have used optogenetics to better understand the function of the amygdala in the brain and behavior, with a particular focus on how these studies have elucidated better understandings of the systems regulating emotion, fear, and memory as well as of the functional neural circuits that involve the amygdala.

## Amygdala and emotion

Perhaps the oldest line of research on the amygdala’s role in the brain originates with how the amygdala regulates emotion and emotional output. Indeed, early work showed that amygdala lesions in monkeys produced the now-classic Kluver-Bucy syndrome (Kluver and Bucy, 1937; Weiskrantz, 1956). The symptoms of the syndrome involved profound alterations in the monkeys’ emotional behavior, especially those involving fear-based behavior. These early findings have led to a considerable number of studies that have expounded upon this function for the amygdala. Studies in humans have confirmed the findings on amygdala lesions, as selective amygdala lesions also appear to produce deficits in emotion-related behaviors, especially those regarding fear (Adolphs et al., 1994, 2005).

Thus, it is not surprising that studies have harnessed the power of optogenetics to develop an improved understanding for how the amygdala influences emotional behavior. In a series of studies, Kay Tye et al. have made several inroads into understanding amygdala functioning in anxiety and anxiety-related behavior. Tye et al. (2011) used an optogenetic approach to distinguish the roles of the basolateral amygdala (BLA) inputs to the medial vs. lateral portions of the central amygdala (CEA) in such behavior in mice. Indeed, previous work had suggested that the medial CEA and its inputs from the BLA drive anxiety and/or fear-related behaviors, whereas BLA inputs to the lateral CEA provide feed-forward inhibition of the medial CEA (Paré et al., 2004; Ciocchi et al., 2010). To distinguish these pathways, Tye et al. (2011) transduced BLA neurons with either the depolarizing cation channel channelrhodopsin-2 (ChR2) or the hyperpolarizing chloride pump halorhodopsin and illumination was provided to the BLA terminals in the lateral CEA. The expression of the opsins was limited to the pyramidal neurons of the BLA through the use of a CaMKIIα promoter. The results indicated that stimulation and inhibition of the BLA terminals in the lateral CEA reduced and increased, respectively, anxiety in the mice, as measured in different tasks. Moreover, the findings strongly suggested that this effect was due to feedforward inhibition of the medial CEA. Illumination of the BLA cells themselves did not produce the same behavioral effect and sometimes produced the opposite effect. With low light levels, illumination of the terminals did not produce reliable antidromic propagation of action potentials back to the BLA, largely eliminating the concern that such propagation could be responsible for the behavioral effect, though it is clear that optical stimulation of axon terminals can produce reliable antidromic propagation to cell bodies (Jennings et al., 2013). These results not only provided a clearer understanding of the amygdala circuits driving behavior but illustrated the importance of targeting specific downstream projection targets, as stimulating or inhibiting specific projections may produce rather different effects than might be found with stimulating or inhibiting the entire structure.

Since this initial work, Tye et al. have extended their focus on anxiety by examining the projections from the BLA to the ventral hippocampus in the regulation of anxiety-related behaviors (Felix-Ortiz et al., 2013). Previous studies had indicated that the BLA projects to the ventral hippocampus and that the ventral hippocampus is also involved in anxiety (Pitkänen et al., 1995, 2000; Bannerman et al., 2003). Using an open-arm plus maze and an open-field chamber, Felix-Ortiz et al. found that inhibition of BLA afferents to the ventral hippocampus, via activation of halorhodopsin, decreased anxiety-related behaviors. Similarly, stimulation of such afferents, using 20 Hz light pulses to activate ChR2, increased anxiety-related behaviors. Importantly, control experiments demonstrated that the effects of the BLA terminal stimulation were not due to antidromic stimulation of the BLA itself or orthodromic stimulation of fibers of passage. Moreover, electrophysiology analysis suggested that the effects of stimulating BLA inputs to the ventral hippocampus were mediated through local circuit mechanisms involving both direct activation of principal cells in the hippocampus and indirect recruitment of inhibitory neurons. In other work, Felix-Ortiz and Tye (2014) examined optical stimulation and inhibition of the BLA axon terminals in the ventral hippocampus during tests of social behavior. In a resident-juvenile-intruder test, stimulation of these terminals, using 20 Hz light pulses, reduced social interactions of the resident, whereas inhibition of these terminals increased such interactions, suggesting that activation of this pathway increased anxiety in the animals whereas silencing of this pathway produced the opposite effect. The effects with stimulation were blocked with microinjections of glutamate receptor antagonists into the ventral hippocampus, indicating that the results were due to stimulation of BLA axon terminals and the concomitant release of glutamate from those terminals. Similar behavioral effects with stimulation were observed with a three-chamber sociability test. Together, the studies by Tye et al. have produced a wealth of knowledge regarding how the amygdala, and especially the BLA, influences anxiety-related behavior through different outputs to other regions. Moreover, the findings have also demonstrated the potential for using optogenetics to develop an improved understanding of the circuits underlying behavior.

Other work has focused on how inputs from the hypothalamus regulate amygdala activity and fear-related behaviors. Previous work had indicated that oxytocin exerts its effects on a variety of behaviors, at least in part, through activation of oxytocin receptors in the CEA (Viviani et al., 2011). However, while oxytocin neurons are located in the hypothalamus, it has not been clear whether the oxytocin is released via dendritic mechanisms and then spreads passively to the amygdala or is released via classic axonal mechanisms (Landgraf and Neumann, 2004). To address this issue, Knobloch et al. (2012) transduced hypothalamic oxytocin neurons using an oxytocin promoter. Optical stimulation of these neurons’ axonal terminals in the CEA produced oxytocin-dependent effects on CEA neuronal activity, indicating that the hypothalamic neurons are able to directly release oxytocin from their axon terminals. In particular, optical stimulation appeared to increase neuronal activity in the lateral CEA and inhibit activity in medial CEA neurons. Anatomical analysis found that the hypothalamic inputs terminated in the lateral, but not medial, CEA. A separate experiment providing stimulation during a test for contextual fear conditioning found an attenuation of freezing behavior with the optical stimulation. Indeed, the overall evidence from this work suggests that the hypothalamic oxytocin neuronal input to the CEA makes synaptic contacts on the lateral, rather than medial, aspect of the CEA, consistent with the function of the lateral CEA in inhibiting medial CEA output and reducing the expression of fear and fear-related behavior. On a larger level, these findings contribute to our understanding of how specific inputs, and even genetically distinct inputs, to the amygdala regulate both behavior and local circuit activity.

## Amygdala and memory

Other work has focused on the use of optogenetics to understand the relationship between the amygdala and memory, especially aversive learning such as fear conditioning. One of the earliest uses of optogenetics in studies of the amygdala examined whether optogenetic stimulation of the lateral amygdala, combined with tones, produces fear conditioning (Johansen et al., 2010). In these experiments, the authors targeted the pyramidal cells of the lateral amygdala. These early findings confirmed that different frequencies of light produced robust firing in lateral amygdala neurons, though with the early version of ChR2 used, the fidelity of the neurons’ responding to 50 Hz light pulses was not as good as that found in response to 20 Hz light pulses. Nonetheless, with 20 Hz stimulation, there was a strong c-fos response in the neurons. Optical stimulation, paired with a tone, produced an increase in freezing both during training and in a later retention test. However, the authors noted that the levels of freezing were considerably lower than had been found in previous studies, an effect that the authors suggest indicates that other mechanisms must also be involved in order to produce full fear conditioning. These findings contributed to previous work showing that activity in the lateral amygdala is critical for the normal development and retention of tone fear conditioning. Moreover, through the use of optogenetics, these experiments were able to selectively target the pyramidal cells while providing temporally precise stimulation, setting the stage for future experiments to build on these early results and develop a clearer understanding of the amygdala’s role in fear conditioning and memory.

Our own work has used an optogenetic approach to examining the role of the BLA in modulating memory consolidation for inhibitory avoidance, a similar aversive learning task (Huff et al., 2013). Prior work suggested that BLA activity in the gamma frequency range (35–45 Hz) is important for synchronizing activity in downstream structures and promoting the consolidation of learning (Bauer et al., 2007; Popescu et al., 2009). However, such work has depended on physiological recordings, which cannot determine whether driving activity in that range alters memory consolidation. Therefore, in our experiments, the BLA pyramidal neurons were transduced with ChR2(E123A), a “ChETA” version of channelrhodopsin that permits high-fidelity responding to light pulses up to 200 Hz (Gunaydin et al., 2010; Yizhar et al., 2011). We found that stimulating the BLA pyramidal cells with bursts of gamma-frequency light pulses (40 Hz) for 15 min immediately after inhibitory avoidance training enhanced retention 2 days later (Huff et al., 2013). Stimulation with bursts of 20 Hz pulses did not produce a significant effect on retention. Previous work has shown that other types of posttraining stimulation (e.g., electrical) of the amygdala produce an inverted-U curve with regard to retention (Gold et al., 1975), but it is not known whether optical stimulation of the BLA also produces such an effect. In another groups of rats, we inhibited BLA neuronal activity immediately after training via activation of the outward proton pump ArchT and found that 15 min of neuronal inhibition, but not 1 min, impaired retention of the learning (Huff et al., 2013). These findings indicated that BLA stimulation in the gamma frequency range enhances memory consolidation and, critically, have provided a set of effective parameters for using optogenetic approaches to modulate memory consolidation that we are continuing to use in new experiments addressing BLA interactions with efferent brain regions.

Other work has investigated a subpopulation of BLA neurons to examine its role in fear conditioning (Jasnow et al., 2013). Specifically, Jasnow et al. targeted the glutamatergic pyramidal cells found in the BLA by driving ChR2 expression with the Thy1 promoter, which limits expression to a specific subpopulation of glutamatergic cells in the BLA and other forebrain regions (Sugino et al., 2006). Optical stimulation of this specific class of BLA neurons during tone fear conditioning impaired retention of the learning while having no effect on the expression of freezing itself during the conditioning (Jasnow et al., 2013). Moreover, optical stimulation of the neurons paired with the tone alone during extinction training enhanced the retention of the extinction learning, again without having any effect on the freezing itself during the extinction training. Generally, it has been thought that activity in the lateral amygdala and medial CEA drive the expression of fear, but electrophysiological characterization of this subpopulation suggests that it shunts activity in lateral amygdala neurons and inhibits activity of medial CEA neurons. Indeed, optical activation of this neuronal subpopulation had no effect on the acute expression of fear but, rather, appeared to influence consolidation specifically for memories that *oppose* fear conditioning.

Recent work has also investigated the interactions of distinct subpopulations within the BLA with efferent targets in relation to fear conditioning. Prior work has noted the existence of neurons within the basal nucleus of the BLA that are responsive to cues associated with footshocks and other neurons that are responsive to cues previously associated with footshocks that have been extinguished. Senn et al. (2014) investigated whether such neurons also show distinct projection patterns to the medial prefrontal cortex (mPFC). To perform a functional investigation of these subpopulations of neurons, retrogradely transported viruses containing Cre-recombinase were injected into either the prelimbic (PL) or infralimbic (IL) cortex while a conditional viral vector expressing the opsins in a Cre-dependent manner was injected into the BLA. As a result, the opsins were selectively expressed in the IL-projecting neurons or the PL-projecting neurons, enabling illumination of the entire BLA to only stimulate or inhibit the specific subpopulation of neurons. Consistent with previous work suggesting a dichotomy between the dorsal regions of the mPFC (PL) and the ventral regions (IL) with regard to fear conditioning (Peters et al., 2009), the authors demonstrated that BLA neurons projecting to the PL are activated by unextinguished cues whereas those projecting to the IL are activated by extinguished cues. Moreover, inhibition of IL-projecting neurons during cue extinction training produced a significant impairment in the retention of the extinction learning, compared to stimulating such neurons. Conversely, inhibition of the PL-projecting neurons during such training enhanced the retention of the extinction learning, compared to stimulating such neurons. While much previous research has focused on the mPFC inputs to the BLA in regulating fear extinction and expression, these findings provide evidence that BLA inputs to distinct mPFC regions also differentially influence such behaviors.

## Amygdala and reward and addiction

Considerable evidence indicates that the amygdala is involved in reward and addiction-related behaviors (Everitt et al., 1999; See et al., 2003; Di Ciano and Everitt, 2004). As part of understanding the precise role played by the BLA in such behaviors, Stuber et al. (2011) examined the pathway from the BLA to the nucleus accumbens (NA) core in modulating and driving reinforcement of operant behavior. Although previous evidence had suggested that the BLA could directly influence dopamine release in the NA (Howland et al., 2002), a key mechanism for reinforcement, it was not clear whether activity in the BLA inputs to the NAcore could actually reinforce operant behavior or was necessary for reward seeking. Stuber et al. found that mice engaged in an operant behavior (nose pokes) in order to receive optical stimulation (burst of 20 Hz light pulses) of the BLA axon terminals in the NAcore. Similarly, optical inhibition, via activation of halorhodopsin, of the BLA terminals decreased responding for a sucrose reward. Strikingly, optical stimulation of mPFC inputs to the NAcore did not produce the same effects. Other work has shown that optical stimulation of BLA inputs to the NAshell are also reinforcing (Britt et al., 2012), though, in contrast to the mPFC inputs to the NAcore, stimulation of mPFC inputs or ventral hippocampal inputs to the NAshell was also found to be reinforcing. Of particular interest in this study was that the authors used the ability to selectively control different glutamatergic inputs to the NAshell, via illumination of opsin-transduced axons from different regions, to determine the degree of plasticity in the connections following cocaine exposure. Together, these findings illustrate not only the increasing evidence for a role for BLA glutamatergic inputs to the NA in regulating reinforcement and reward but also that, depending on the part of the NA targeted, other glutamatergic inputs do not necessarily produce identical results.

Other studies have indicated that, consistent with its role in different forms of associative learning, the BLA is a key part of the neural circuit that drives drug-seeking behavior triggered by drug-associated cues (See et al., 2003). However, as the BLA projects to both the PL and the NAcore, both of which are considered critical components of a general drug-seeking circuit (McFarland and Kalivas, 2001; McFarland et al., 2004; LaLumiere and Kalivas, 2008), it has not been clear which pathway is responsible for driving cue-induced drug-seeking. Recent work by Stefanik and Kalivas (2013) has used optogenetics to investigate this issue. The BLA was transduced with ArchT. Inhibition of BLA terminals in either the PL or NAcore reduced cue-induced reinstatement of cocaine-seeking, indicating that activity in both pathways is obligatory for associative cue-driven cocaine-seeking.

## Circuitry of the amygdala and interactions with other brain regions

A number of studies have used optogenetic approaches to develop a better understanding of the functional connections between the amygdala and other brain regions. For example, Li et al. (2012) have investigated the role of kappa opioid receptor signaling in the bed nucleus of the stria terminalis (BNST). Patch-clamp recordings in the BNST provided evidence that activation of kappa opioid receptors inhibits GABAergic transmission via presynaptic mechanisms. As the CEA provides an important GABAergic input to the BNST, one that has been implicated as a critical pathway in the central stress system (Jasnow et al., 2004; Walker and Davis, 2008), the authors used optogenetics to target and control activity in this pathway. The results indicated that kappa opioid receptor activation inhibited GABAergic transmission in this pathway specifically, a result that would have been difficult to demonstrate using other techniques.

Other research has used optogenetics to delineate precisely how the amygdala influences activity in other regions. Luna and Morozov (2012) contrasted the microcircuitry of BLA inputs vs. anterior piriform inputs to the posterior piriform cortex. Although both structures were found to innervate deep pyramidal cells of the posterior piriform, the BLA and anterior piriform connected with different kinds of interneurons. Specifically, the BLA produced strong connections with fast-spiking interneurons, whereas the anterior piriform had its strongest synapses on irregular-spiking interneurons. As these different classes of interneurons synapse on different regions of the pyramidal cells (somatic vs. distal dendritic), the feedforward inhibition from BLA vs. anterior piriform inputs would be expected to have profoundly different effects on the likelihood of spiking in the principal cells of the posterior piriform.

Several studies have used optogenetics to understand amygdala function and interactions in combination with a variety of other techniques, an approach that will likely dominate much future work as optogenetics affords significant advantages in our ability to gain deeper knowledge regarding neural circuitry. For example, experiments have focused on the well-known connections between the BLA and the mPFC. The reciprocal connections between these regions appear to be involved in a wide variety of behavioral and higher cognitive functions. Yet, the mPFC receives inputs from many other structures and the distinctions among the connections formed by these inputs have not been clear. Therefore, Little and Carter (2012) investigated how BLA, ventral hippocampal, midline thalamus, and contralateral mPFC inputs to the layer two pyramidal neurons of the mPFC make synaptic connections. The authors used optogenetics to target specific pathways by transducing the efferent structures with ChR2 and providing illumination to their axonal terminals in the mPFC. Moreover, they combined their optogenetic manipulations with two-photon microscopy in order to determine the functional connections on a subcellular level. The results from this study indicate that the different regions do, in fact, make different subcellular connections. The BLA appears to make synaptic connections much closer to the soma, relative to other regions, especially the thalamic inputs. Additionally, the BLA inputs target spines of an “intermediate” size, along with ventral hippocampal inputs, in contrast to the thalamic inputs to the large spines and the contralateral mPFC inputs to the small spines. As both the size of the spine and the distance from the soma govern the relative strengths of the inputs, these findings shed light on how different regions influence local circuit activity in other regions. In a follow-up study, Little and Carter (2013) extended their findings, again using optogenetics combined with two-photon microscopy. In this case, their findings indicated that BLA inputs to the mPFC were considerably stronger on mPFC neurons that innervated the BLA, compared to mPFC neurons that provide inputs to the contralateral mPFC. Together, these findings have contributed to a deeper understanding for how the BLA and mPFC interact and, critically, provide a foundation for understanding how BLA inputs to the mPFC may regulate PFC activity and PFC-dependent functions.

Work has examined other brain regions’ inputs to the amygdala. In a recent study, Carter et al. (2013) found evidence of a circuit involving projections from the parabrachial nucleus in the brainstem to the CEA that suppresses appetite. After genetically identifying and targeting neurons in the parabrachial nucleus to determine their ability to suppress appetite in mice, the authors then transduced these cells with ChR2 and provided illumination to downstream targets. Although stimulating parabrachial axon terminals in the BNST had no effect on food intake, stimulation between 20–40 Hz of the axon terminals in the lateral CEA reduced food consumption. In contrast to many studies that have used 20 Hz stimulation but consistent with our own work (e.g., Huff et al., 2013), Carter et al. found that 20 Hz stimulation did not produce as strong a behavioral effect as found with the 30 and 40 Hz stimulation. By utilizing both the genetic targeting ability of combining optogenetics with transgenic mice and by targeting the axon terminals, these findings provide a significant step forward in understanding how genetically distinct neuronal populations connect with different regions in the brain and, in turn, regulate appetite-related behavior.

Optogenetic studies have also targeted specific interneuron populations in the BLA to understand local circuits. Chu et al. (2012) examined how dopamine influences parvalbumin-positive interneurons in the BLA and, thereby, influence principal cell activity. This issue is of importance because previous work has shown that dopamine influences BLA activity and modulates memory consolidation (Bissiere et al., 2003; LaLumiere et al., 2004, 2005). Prior work has found that D2 receptor activation in the BLA suppresses feedforward inhibition, thereby providing a gating mechanism for synaptic plasticity in the amygdala (Bissiere et al., 2003), while other work has also found that dopamine disinhibits the BLA via inhibition of intercalated cells in a D1 receptor-dependent manner (Marowsky et al., 2005). Using a Cre line of transgenic mice, Chu et al. were able to target ChR2 expression to the parvalbumin-positive cells of the BLA, which are believed to be the major class of interneurons in the structure. The authors then demonstrated that dopamine selectively reduced GABAergic transmission to principal cells, but not to other interneurons, and that this occurred in a presynaptic D2 receptor-dependent mechanism. These findings provide additional confirmation of the critical role of dopamine, and especially D2 receptors, in modulating BLA activity. Moreover, these findings were demonstrated in a specific subclass of interneurons, an important issue as other work has suggested that different stimuli influence different subtypes of interneurons in the BLA (Bienvenu et al., 2012).

## Conclusions

As a heterogeneous collection of nuclei with a variety of influences on neural functioning and behavior, the amygdala has been the subject of countless neurobiological studies. Our ability to investigate this structure and its role in the brain and behavior has been significantly altered by the development of optogenetics. The studies reviewed here and listed in Table 1 have provided an understanding for the kinds of questions that this approach can address with regard to the amygdala and, critically, have provided roadmaps for continuing research into amygdala functioning. The findings from these studies indicate that the local circuitry in the amygdala regulates a number of emotion-related behaviors and have produced a clearer picture for how this circuitry works. Other work has begun providing glimpses into how the BLA influences activity in efferent structures, even delineating the local microcircuits to determine the precise differences between BLA inputs to the region compared to other inputs. Moreover, other studies presented here have demonstrated that specific frequencies of BLA activity differentially influence behavior and memory consolidation. These findings will prove invaluable as neuroscience builds a deeper understanding of the full connectivity of the nervous system on a functional level. All of this work and the findings from these experiments, however, would not have been possible without the temporal, spatial, and/or genetic precision afforded by optogenetics. As future studies utilizing optogenetic approaches build upon these early findings, we should expect considerable advancement in our understanding of amygdala functioning in behavior and in interactions with other brain systems.

**Table 1:** **Description of reviewed publications using optogenetics in investigations of the amygdala**.

**Publication**	**Area of investigation**
Tye et al. (2011)	Anxiety: Stimulation/inhibition of BLA inputs to the lateral CEA reduce/increase anxiety-related behavior in mice
Felix-Ortiz et al. (2013)	Anxiety: Stimulation/inhibition of BLA inputs to the ventral hippocampus increase/decrease anxiety-related behavior in mice
Felix-Ortiz and Tye (2014)	Social behavior: Stimulation/inhibition of BLA inputs to the ventral hippocampus reduce/increase social behaviors
Knobloch et al. (2012)	Fear-related behavior: Hypothalamic oxytocin neurons projections to the lateral CEA in regard to their role in altering medial CEA activity and freezing behavior
Johansen et al. (2010)	Fear conditioning and memory: Pairing of optical stimulation of lateral amygdala neurons with tones produce fear conditioning
Huff et al. (2013)	Memory: Posttraining optical stimulation and inhibition of BLA glutamatergic neurons enhance/impair memory consolidation for inhibitory avoidance
Jasnow et al. (2013)	Fear conditioning and memory: Subpopulation of glutamatergic neurons in the BLA oppose fear conditioning and enhance extinction of fear conditioning
Senn et al. (2014)	Fear conditioning and memory: BLA projections to the IL and PL of the mPFC have opposing roles in the extinction of fear conditioning
Stuber et al. (2011)	Reward: BLA inputs to the NAcore reinforced operant behavior
Britt et al. (2012)	Reward: BLA inputs to the NAshell reinforced operant behavior
Stefanik and Kalivas (2013)	Addiction: Inhibition of BLA inputs to the NAcore or PL reduce cue-induced cocaine-seeking
Li et al. (2012)	Circuitry: Activation of kappa opioid receptors inhibits GABAergic transmission from the CEA to the BNST
Luna and Morozov (2012)	Circuitry: Microcircuitry of BLA inputs to posterior piriform cortex
Little and Carter (2012)	Circuitry: Differences in BLA inputs to the mPFC, compared to inputs from other regions, in terms of subcellular connections
Little and Carter (2013)	Circuitry: Differences between BLA inputs to mPFC neurons that innervate the BLA and those that innervate the contralateral mPFC
Carter et al. (2013)	Circuitry: Stimulation of parabrachial nucleus projections to the CEA suppress appetite
Chu et al. (2012)	Circuitry: Dopamine reduces GABAergic transmission from parvalbumin-positive interneurons to principal cells in the BLA

## Conflict of interest statement

The author declares that the research was conducted in the absence of any commercial or financial relationships that could be construed as a potential conflict of interest.
